# Land-based crop phenotyping by image analysis: consistent canopy characterization from inconsistent field illumination

**DOI:** 10.1186/s13007-018-0308-5

**Published:** 2018-05-26

**Authors:** Joshua Chopin, Pankaj Kumar, Stanley J. Miklavcic

**Affiliations:** Phenomics and Bioinformatics Research Centre, University of South Australia, Mawson Lakes, 5095 Australia

**Keywords:** Wheat, Colour, Phenomics, NDVI

## Abstract

**Background:**

One of the main challenges associated with image-based field phenotyping is the variability of illumination. During a single day’s imaging session, or between different sessions on different days, the sun moves in and out of cloud cover and has varying intensity. How is one to know from consecutive images alone if a plant has become darker over time, or if the weather conditions have simply changed from clear to overcast? This is a significant problem to address as colour is an important phenotypic trait that can be measured automatically from images.

**Results:**

In this work we use an industry standard colour checker to balance the colour in images within and across every day of a field trial conducted over four months in 2016. By ensuring that the colour checker is present in every image we are afforded a ‘ground truth’ to correct for varying illumination conditions across images. We employ a least squares approach to fit a quadratic model for correcting RGB values of an image in such a way that the observed values of the colour checker tiles align with their true values after the transformation.

**Conclusions:**

The proposed method is successful in reducing the error between observed and reference colour chart values in all images. Furthermore, the standard deviation of mean canopy colour across multiple days is reduced significantly after colour correction is applied. Finally, we use a number of examples to demonstrate the usefulness of accurate colour measurements in recording phenotypic traits and analysing variation among varieties and treatments.

**Electronic supplementary material:**

The online version of this article (10.1186/s13007-018-0308-5) contains supplementary material, which is available to authorized users.

## Background

The century-old [1] endeavour to understand complex genotype $\times$ environment (G $\times$ E) interactions and their effect on plant phenotype has triggered a series of breakthroughs in the field of plant phenotyping. Manual methods for recording phenotypic traits gradually evolved [2] to semi-manual and automatic methods, through the use of electronic hand-held devices and imaging equipment. Automated phenotyping through image capture and analysis was first achieved with state of the art robotics and imaging sensors in controlled environments [3–7]. More recently, in order to further understand G $\times$ E interactions, the ‘environment’ side of the equation has increased in accuracy thanks to phenotypic analysis in the field [8–11].

The range of phenotyping that can occur in the field is vast. Hardware solutions that have been proposed range from aerial imaging [12, 13], large and expensive purpose-built systems [14, 15], small and expensive purpose built systems [16–18] to small and inexpensive systems [19, 20]. While each approach possesses distinct advantages and disadvantages, the increase in throughput and volume of high resolution RGB (Red, Green and Blue channel) data offered by imaging of field trials comes at a cost: new methodologies are required to overcome new challenges. In this article the challenge of accurately recording plant colour under varying illumination conditions in the field is considered. In essence, the critical question is how is one to know from consecutive images alone if a plant has become darker over time, or if the weather conditions have simply changed from clear to overcast? Despite colour being reported as an important plant phenotypic trait [2, 21, 22] indicative of plant health, an early indicator of a state of stress or disease, and related to plant chlorophyll [23, 24] and nitrogen content [25], it has been largely neglected in the literature until recently [14, 15, 26].

Existing approaches to compensate for variations in illumination can be classified as hardware or software solutions. Utilising the camera’s hardware, a common approach is to simply use the ‘automatic-exposure’ mode on digital cameras [14, 15, 27], which makes use of the camera’s in-built metering system to automatically decide on an appropriate shutter speed and aperture. This approach comes with a number of drawbacks such as spherical aberration [28] and a lack of consistency in recording colour. From a theoretical standpoint the computer vision community has proposed many post-processing techniques to determine the illuminant in an image, which they call the colour constancy problem. For example, Finlayson et al. [29] used histogram equalization to provide illumination invariance across devices and before that calculated individual likelihoods that each possible scene illuminant was illuminating the test image [30]. Forsyth [31] estimated the illuminant in images using surface reflection functions and D’Zmura et al. [32] showed that in an arbitrary scene the chromacity of reflected light from at least four surfaces is required to estimate illumination.

In the case of field-based phenotyping, most image analysis approaches taken to the illumination challenge focus on accurately determining canopy coverage or other traits, rather than correcting the canopy colour itself. For example, in [33] the authors propose a method for image segmentation that is invariant to illumination. However, the segmented plant still contains the illuminated pixels. In [34] the authors train two separate support vector machines, for high and low luminance, then propose an approach to classify every image as one of those two, before applying the respective SVM for segmentation. Both of these examples are useful for approximating coverage, yet provide misleading colour information. In a study closely related to the present work, Grieder et al. [35] made use of a colour checker for standardizing colour images over a day but only used a linear transformation to correct the colour values. We will show that for images in the field a quadratic model is more accurate. Furthermore, as that study was focussed on assessing canopy coverage of plots, an investigation into the accuracy of colour measurements over time and how robust they are to varying illumination conditions was not within the scope of their article.

In this paper we propose the use of an X-rite Colour Checker chart [36] and a quadratic model for correcting the colour of plant images taken in the field. Two standard digital cameras and a colour chart are mounted on an inexpensive, land-based vehicle in order to capture a time series of high resolution images of individual plots of a wheat field over a period of four months. The details of this field trial and the construction of the vehicle are explained in the Methods section. An image processing pipeline for analysing the images, comprised of pre-processing and segmentation stages, is also provided in this section. A quadratic model is then used to ascertain the relationship between observed and reference colour chart values for every image and the appropriate transformation is applied to each of them. In the Results section we demonstrate the effectiveness of the quadratic model and produce evidence of its robustness over multiple days. Finally, we provide examples illustrating the application of accurate colour measurement by studying variations across varieties and treatments, as well as predicting the normalized difference vegetation index of plants using mean canopy colour.

## Methods

The field trial was conducted at Mallala (− 34.457062, 138.481487), South Australia, in a randomized complete block design with a total of 60 plots consisting of ten spring wheat (*Triticum aestivum L.*) varieties and six replicates for each. To mitigate the effects of border rows, an additional plot (not included in the analysis) was planted at the beginning and end of each row of plots. Plots were 1.2 m wide and 4 m long with a gap of approximately 1 m between columns and rows. Half of the replicates were treated with nitrogen at a standard rate of 80 kg nitrogen, 40 kg phosphorus and 40 kg potassium per hectare and the other half received no treatment. The macronutrients nitrogen, phosphorus and potassium were first applied on 12 August 2016 and imaging of the plots took place between August 23 and November 18 of the same year. A detailed report on the outcome of this field experiment is the subject of a separate communication.

**Fig. 1:**
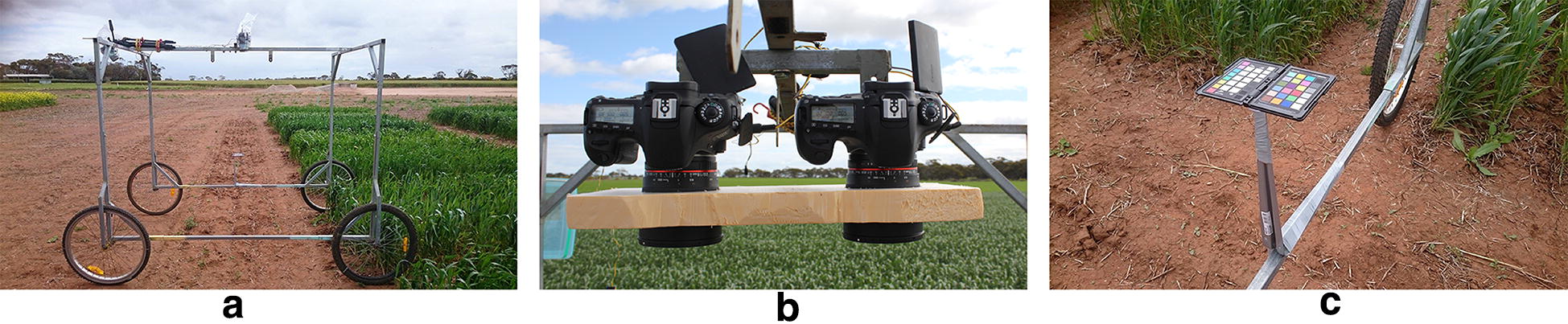
Imaging wagon. **a** The ground based vehicle for imaging in the field. **b** Two stereo cameras are placed in the centre of the top section, with a third camera at an oblique view not used in this experiment. **c** An x-rite colour checker is also placed on the side of the vehicle, visible by the camera on the same side

The ground-based vehicle used for image capture is shown in Fig. 1a. This ‘wagon’ is comprised of a steel frame and four wheels with a central overhead rail for mounting imaging sensors. While capable of housing a third camera from an oblique view, only the central stereo pair of cameras, shown in Fig. 1b, was used for this experiment. An X-rite colour checker was attached to the left side of the wagon, shown in Fig. 1c, which was always visible from the viewpoint of the camera on the corresponding side. The specifications of the chart and motivation for using it are outlined in the Colour Correction section.

Images were acquired using a stereo pair of Canon EOS 60D digital cameras, placed approximately 20 cm apart and 2 m above ground level. Manual focus was used during all imaging sessions with cameras focused at 2 and 1.5 m during early and late plant growth stages, respectively. Camera settings were as follows; focal length 18 mm, aperture f/9.0, ISO—automatic and exposure time 1/500 s. Cameras were temporally synchronized to capture images within 1 ms of each other.

### Image processing

**Fig. 2:**
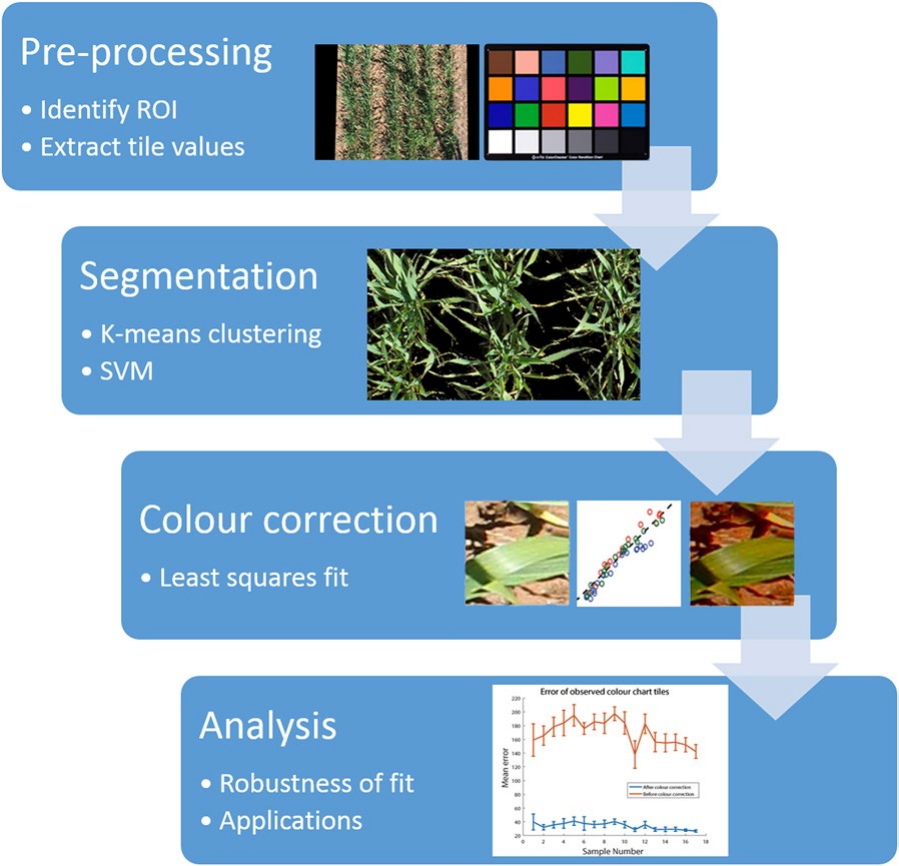
Image analysis pipeline. The images are pre-processed to extract the region of interest and the values of colour checker tiles before plant pixels are segmented from the background. A least squares approach is then used to fit a quadratic to the colour data and correct the plant pixels. Finally, the corrected mean canopy colour can be extracted from the segmented image and used for analysis

All image processing and analysis steps were conducted in MATLAB 2017a. A flowchart outlining each step of the image analysis pipeline is shown in Fig. 2. While this section provides an overview of each image processing task associated with analysing images from the field, a more detailed explanation can be found in Additional file 1.

Before applying the colour correction technique to images from the field two important pre-processing steps were taken. Both of these steps are necessary due to challenges arising from field conditions. The two pre-processing steps are detecting the region of interest (ROI), the image region containing plant pixels, and extracting the colour checker. To provide consistency in the analysis of images of all plant varieties and throughout the season, with the added benefit of avoiding spurious contributions from weeds present in the images but lying outside the ROI, the ROI was chosen to be all pixels inside the parallel rails of the phenotyping vehicle. From an image processing perspective this involves detecting the two rails, using a Hough transform [37], and creating a mask the same size as the original image which, after the application of a Hadamard product [38], removes the background regions outside of the ROI perimeter (Additional file 1: Fig. S1). The colour chart is extracted using a template matching algorithm which iteratively searches the image for regions which resemble a pre-saved generic image of a colour checker.

After image pre-processing the next step is segmentation. Plant pixels in all images were segmented from the background using support vector machines (SVM) [39] which were trained on the output of k-means clustering [40]. SVM is a supervised machine learning technique which, for a set of data with two classes, attempts to find the best hyperplane that separates the two data classes. Using k-means clustering, each training image is segmented into 20 clusters with minimal intra-class variance, then each cluster is given a label as green plant or background. The centre of each cluster, or mean colour, is then used as an individual training data point for the SVM (Additional file 1: Fig. S2). As this process takes far less time than manually segmenting entire images, it allows more total images to be used for training, capturing more variation across plots and over time.

### Colour correction

The goal of colour correction is to transform the pixel values in an image in such a way that the observed values of the colour checker tiles will match their true values after the transformation. This requires constructing a model using the observed values from the colour checker in an image, which can take as input a new colour triplet and output its corrected values. In this article we have chosen to conduct the colour correction stage in the CIEL*a*b* 1976 colour space, abbreviated as L*a*b* hereinafter. The L*a*b* space was chosen as it was designed to be a device-independent space. In addition, through experimentation and following a review of the literature, the L*a*b* space was found to perform better generally than the RGB space for colour correction [41]. While the L*a*b* space was determined to be the most useful for performing colour correction, all results have been converted back to RGB values for illustrative purposes, since the RGB space mimics human vision and each channel contains values in the same range of [0, 255]. Conversion between RGB and L*a*b* values is carried out using the standard formulae [42].

**Fig. 3:**
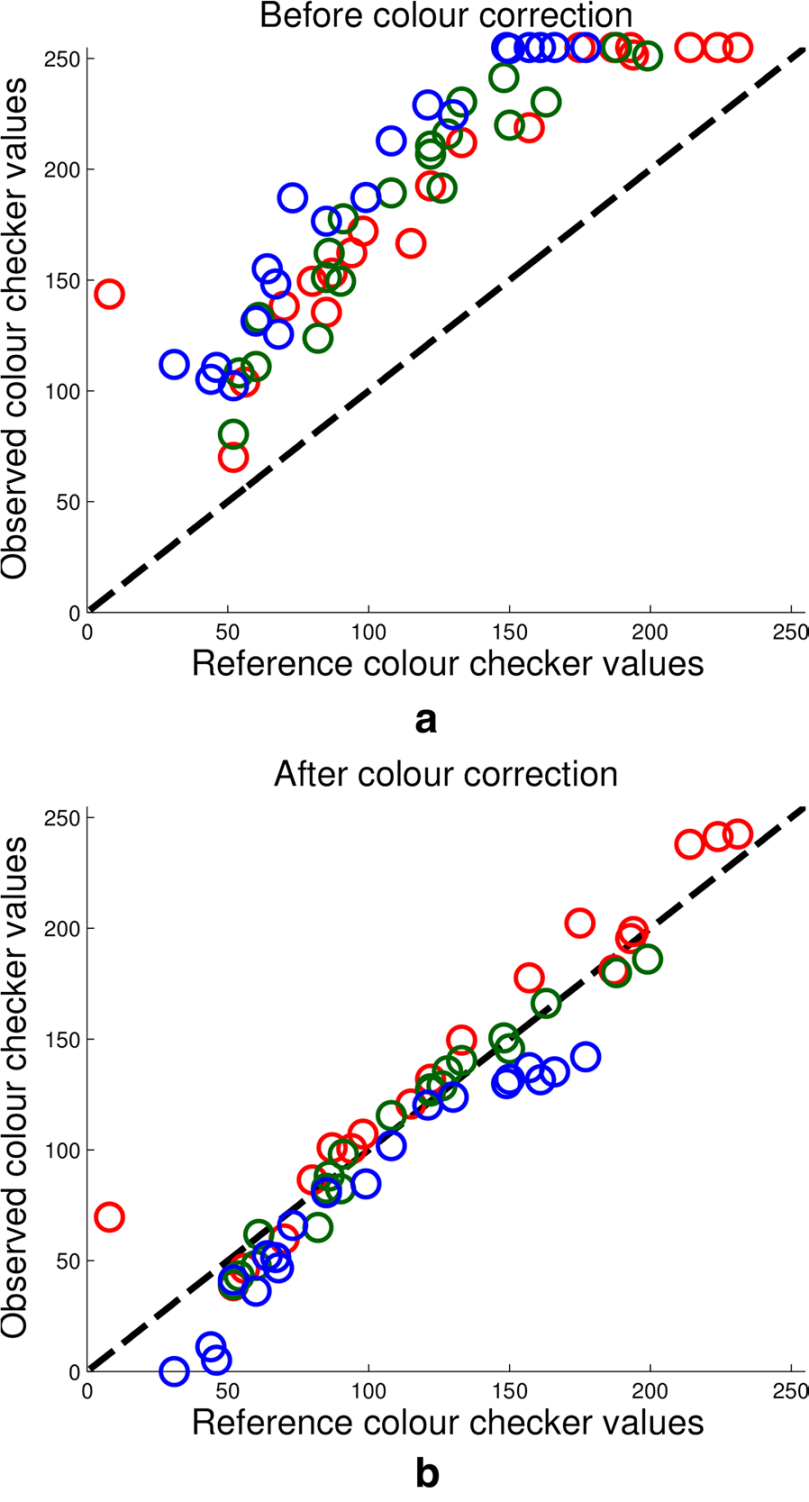
Colour correction. **a** Reference colour checker values vs observed colour checker values before colour correction takes place. **b** The same graph after the colour correction process has taken place

Figure 3a shows the relationship between true and observed RGB values of the 24 colour checker tiles for a typical image. As the relationship does not appear to be linear, a quadratic model was chosen to fit the data. As an aside, this model was used to fit all 24 tiles as well as a number of subsets of tiles, in order to determine whether the range of colours provided by the colour checker was appropriate. We found that using all tiles provided the best results and most versatile model. The quadratic model makes use of all three channels and their squares when predicting colour values. For example, the formulation for the $L*$ channel is as follows,1where $L_i$, $a_i$ and $b_i$ refer to the observed $L*$, $a*$ and $b*$ values of the *i*-th colour checker tile, $\alpha$ refers to the fitting coefficients and $\hat{L}_i$ refer to the $L*$ reference values of the *i*-th tile. The same methodology is used with $\beta$ and $\gamma$ coefficient values for the $a*$ and $b*$ channels, respectively. Finally, the least squares method is used to determine the values of $\alpha$, $\beta$ and $\gamma$. Applying the model to the observed colour checker values shown in Fig. 3a, after correction yields the results shown in Fig. 3b.

## Results and discussion

**Fig. 4:**
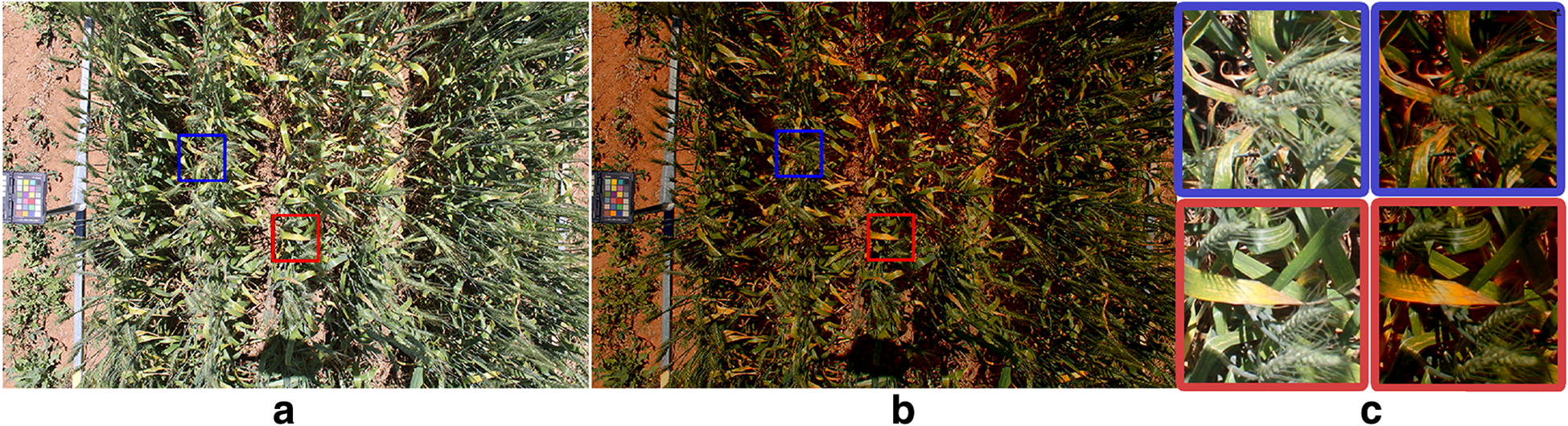
Sample image before and after colour correction. **a** Sample wheat plot image before colour correction. **b** The same wheat plot after colour correction. **c** Magnified sections of each image showing the increased contrast between green and senesced leaves in the corrected image

An example of a colour corrected image of a field plot is shown in Fig. 4. The magnified image regions illustrate the increased contrast between healthy and senesced plant regions in later stages of plant life. We demonstrate the usefulness of colour correction by first illustrating the increase in accuracy and consistency it provides when measuring mean canopy colour in the field. Fixing camera settings and applying colour correction, rather than relying on camera automatic-exposure settings, is preferential for a number of practical reasons which will be explained in this section. However, to prove this point in a quantitative manner, the reproduction error of colours for each approach is calculated. Once it is established that colour corrected images are more useful than uncorrected images we show how they can be used in typical phenotypic analyses. This includes outlining key differences in time series data of mean canopy colour for wheat plots of different varieties under different treatment conditions. Furthermore, we show that colour corrected images can be used to find relationships between the RGB values of plot canopies and their manually measured normalized difference vegetation index (NDVI) values.

### Quantifying the importance of colour correction

**Fig. 5:**
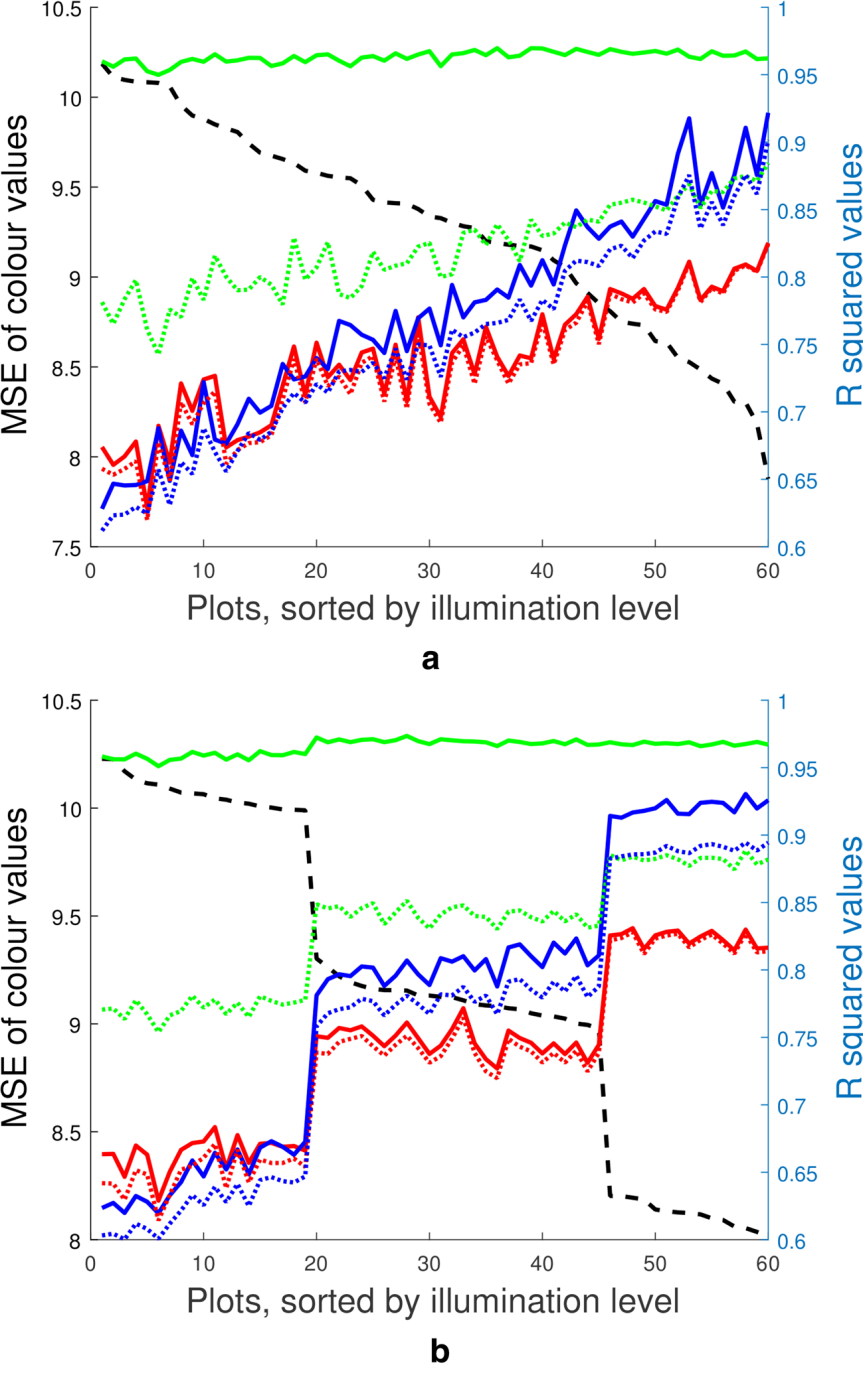
Quadratic fits over a day with **a** constant change in illumination and **b** step-changes in illumination. Graphs displaying the $R^2$ value of a linear fit, (dashed lines) and quadratic fit (solid lines) for the red, green and blue channels of the colour checker tiles. The 60 wheat plots in the experiment are ordered by illumination i.e. the difference between observed and reference colour checker values, shown on the left-hand side *y*-axis and represented by the black dashed line

While most literature concerning image-based phenotyping in the field do not perform colour correction at all, the few that do use a simple linear scaling of pixel values [35]. However, the observed pixel values in images compared to their true values, as shown in Fig. 3, appear to follow a quadratic rather than linear relationship. To compare how well the data is fit by linear and quadratic models, we compare the approach outlined by Grieder et al. with the method presented here. The dashed and solid lines in Fig. 5a, b show the $R^2$ values of linear and quadratic fits of the observed colour values, respectively. The values on the horizontal axis represent the 60 plots of wheat used in the field trial, ordered in order of increasing illumination. That is, the first data point represents the plot with the largest difference between observed and reference colour checker values. The mean square error (MSE) can be seen on the left side horizontal axis and is illustrated by the dashed black line.

Figure 5a, b represent two different days during the experiment, therefore changes in illumination with time will also be different and as such the order of plots is not maintained between figures. The purpose of this choice for visualization is two-fold. First, one can see that on these 2 days the quadratic model always provided a more accurate fit of the data. Furthermore, while the improvement between a linear and quadratic fit is insignificant in the red and blue channels, it is substantial in the green channel. As the defining characteristic of plant pixels is their green intensity, this is the most important channel to correct. Table 1 shows the mean $R^2$ values for fitting a quadratic or linear model to the red, green and blue channels over the 17 imaging sessions. While the average difference in $R^2$ value between a linear and quadratic fit in the red and blue channels is 0.05 and 0.02 respectively, it is a substantially higher 0.12 in the green channel.

The second reason for ordering the data in order of illumination is to highlight how the illumination conditions affect the goodness of fit for these models, and subsequently the accuracy of the final colour correction. The two examples in Fig. 5a, b show that there is a clear inverse relationship between the degree of illumination and how well the two methods were able to fit the colour data in the red and blue channels. The green channel has not only been fit with larger $R^2$ values overall, but appears to also be less affected by illumination conditions when fit with a quadratic model.

**Table 1:** $R^2$ values for linear (L.) and quadratic (Q.) fits of the red (R), green (G) and blue (B) data for 17 imaging sessions (rows), averaged over the 60 plots each time (columns)

	R		G		B	
	L.	Q.	L.	Q.	L.	Q.
1	0.70	0.71	0.79	0.91	0.83	0.86
2	0.75	0.75	0.84	0.96	0.85	0.88
3	0.71	0.72	0.80	0.96	0.81	0.84
4	0.71	0.72	0.80	0.96	0.74	0.76
5	0.65	0.67	0.74	0.94	0.67	0.69
6	0.76	0.76	0.83	0.95	0.76	0.78
7	0.73	0.74	0.82	0.96	0.75	0.77
8	0.73	0.74	0.83	0.97	0.76	0.78
9	0.68	0.69	0.78	0.96	0.64	0.67
10	0.74	0.75	0.84	0.96	0.72	0.73
11	0.72	0.72	0.87	0.97	0.89	0.89
12	0.73	0.74	0.83	0.96	0.76	0.79
13	0.83	0.83	0.89	0.96	0.88	0.89
14	0.82	0.82	0.89	0.96	0.88	0.89
15	0.81	0.81	0.88	0.96	0.89	0.91
16	0.82	0.82	0.88	0.96	0.90	0.92
17	0.84	0.84	0.90	0.96	0.92	0.94

**Fig. 6:**
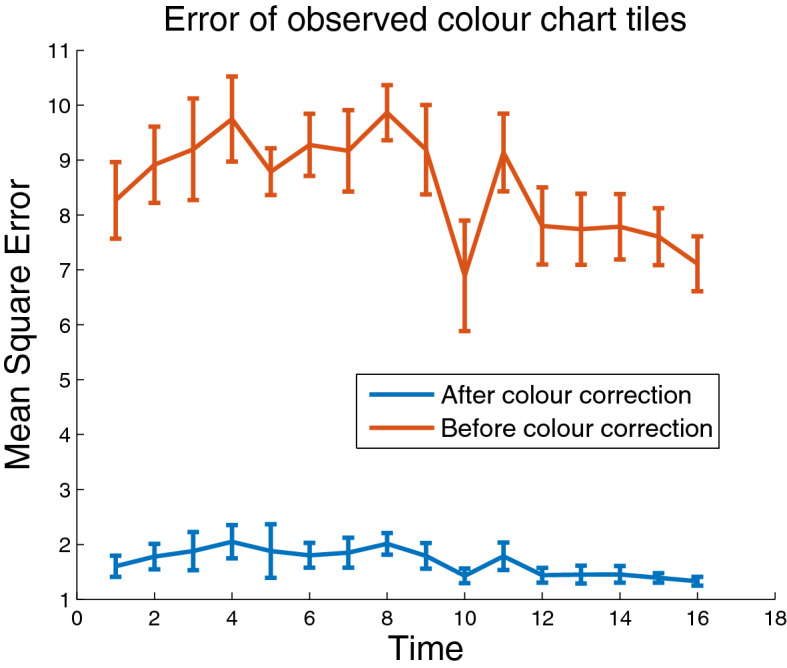
Error of observed colour chart tiles. Error between observed and reference colour chart tile values over 16 imaging sessions for colour corrected images (blue) and original images (red)

Figure 6 shows the mean and standard deviation of error between observed and reference colour checker values, over all 16 imaging sessions, before and after the colour correction process, using the quadratic model. The error, *E*, which is the average Euclidean distance between the two colour triplets or mean square error (MSE), is calculated according to Eq. 2, where *R*, *G* and *B* denote the red, green and blue colour channels respectively, $i=1{:}24$ denotes the 24 colour checker tiles and $\hat{R}_i$ and $R_i$ denote the reference and observed red values of the *i*th tile, respectively, and similarly for G, green, and B, blue. The amount of error after colour correction is approximately four times lower than before it was applied. Furthermore, there is more variation across imaging sessions when colour correction is not applied, as error rates increase and decrease over time with no predictable pattern. This is due to the different illumination conditions and the effect they have on colour correction. Therefore, another meaningful analysis is to investigate how well the colour correction process is able to maintain a consistent measure of colour for each plot across multiple days.

2 $$\begin{aligned} E=\frac{1}{24} \sum _{i=1}^{24} \sqrt{(\hat{R_i}-R_i)^2 + (\hat{G_i}-G_i)^2 + (\hat{B_i}-B_i)^2} \end{aligned}$$

**Fig. 7:**
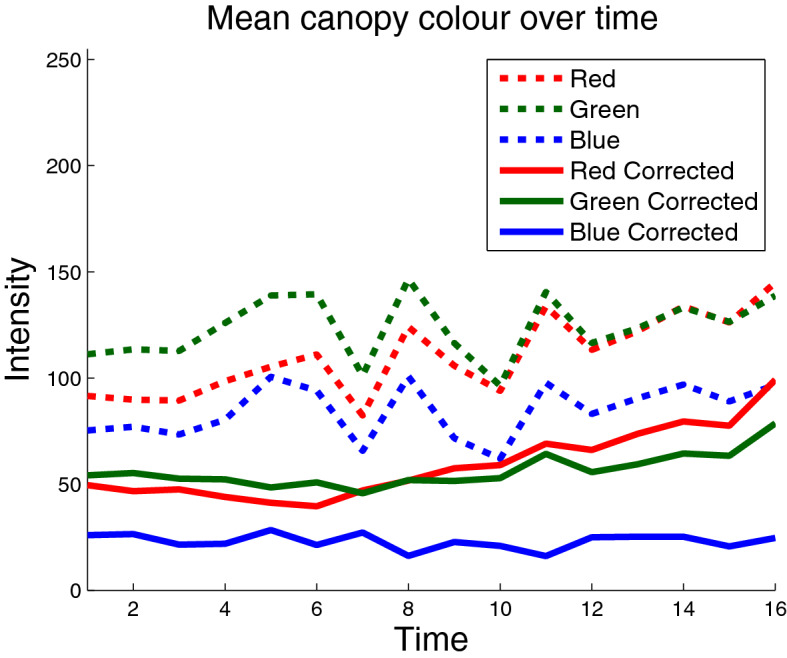
Mean canopy colour over time. Mean canopy colour values in red, green and blue, before (dashed lines) and after (solid lines) colour correction, for a single wheat plot. The *x*-axis represents the 16 samples taken over four months of the experiment

Figure 7 shows mean canopy colour in the red, green and blue channels for a sample wheat plot over all 16 imaging sessions. The purpose of this plot is not to demonstrate the mean intensities themselves but rather to depict the consistency of colour measurements over multiple days with varying levels of illumination. The jagged nature of the dashed lines, colours before correction, implies changes in colour too severe and irregular to be associated with a biological phenomena. Instead, the mean intensities are changing over time primarily due to imaging sessions being conducted on bright or overcast days. On the other hand, the solid lines, representing colour values after correction, appear much more stable over a long period of time. It is also of note that the steady rise in pixel intensities in the colour corrected data toward the end of the experiment is to be expected, as plants are beginning to senesce and turn yellow, which has higher red intensities than green. Table 2 shows the standard deviation of mean canopy colour over the 16 days for all 60 plots in the trial. In no case did the standard deviation of corrected values exceed the standard deviation of uncorrected ones, which were 63% higher, on average.

**Table 2:** Standard deviation of green intensity values over 16 imaging sessions for the 60 plots before (B) and after (A) the application of colour correction

	B	A		B	A		B	A		B	A
1	19	10	16	13	9	31	15	14	46	13	10
2	16	10	17	14	8	32	15	9	47	19	7
3	13	8	18	12	9	33	15	8	48	16	11
4	13	12	19	15	8	34	17	10	49	20	17
5	14	10	20	14	7	35	14	10	50	15	11
6	12	9	21	11	11	36	14	10	51	16	11
7	14	11	22	11	9	37	14	12	52	11	8
8	12	10	23	13	6	38	18	10	53	15	8
9	16	7	24	16	9	39	18	10	54	17	7
10	16	10	25	17	7	40	17	10	55	14	10
11	14	9	26	14	9	41	15	9	56	14	9
12	15	11	27	13	9	42	15	9	57	21	16
13	16	11	28	14	9	43	17	9	58	19	10
14	15	11	29	15	5	44	15	11	59	14	10
15	17	8	30	18	7	45	13	8	60	14	9

### Automatic exposure settings versus colour correction

The automatic exposure mode of standard digital cameras uses the camera’s in-built metering systems to automatically choose values for the aperture and shutter speed parameters. However, allowing the maximum amount of light to hit the camera’s sensor by choosing the minimum safe shutter speed which will avoid motion blurring is preferable to letting the camera choose the shutter speed parameter on a per-image basis. Furthermore, in repeated experiments with a fixed schematic the depth of field, related to the aperture, should remain consistent.

**Fig. 8:**
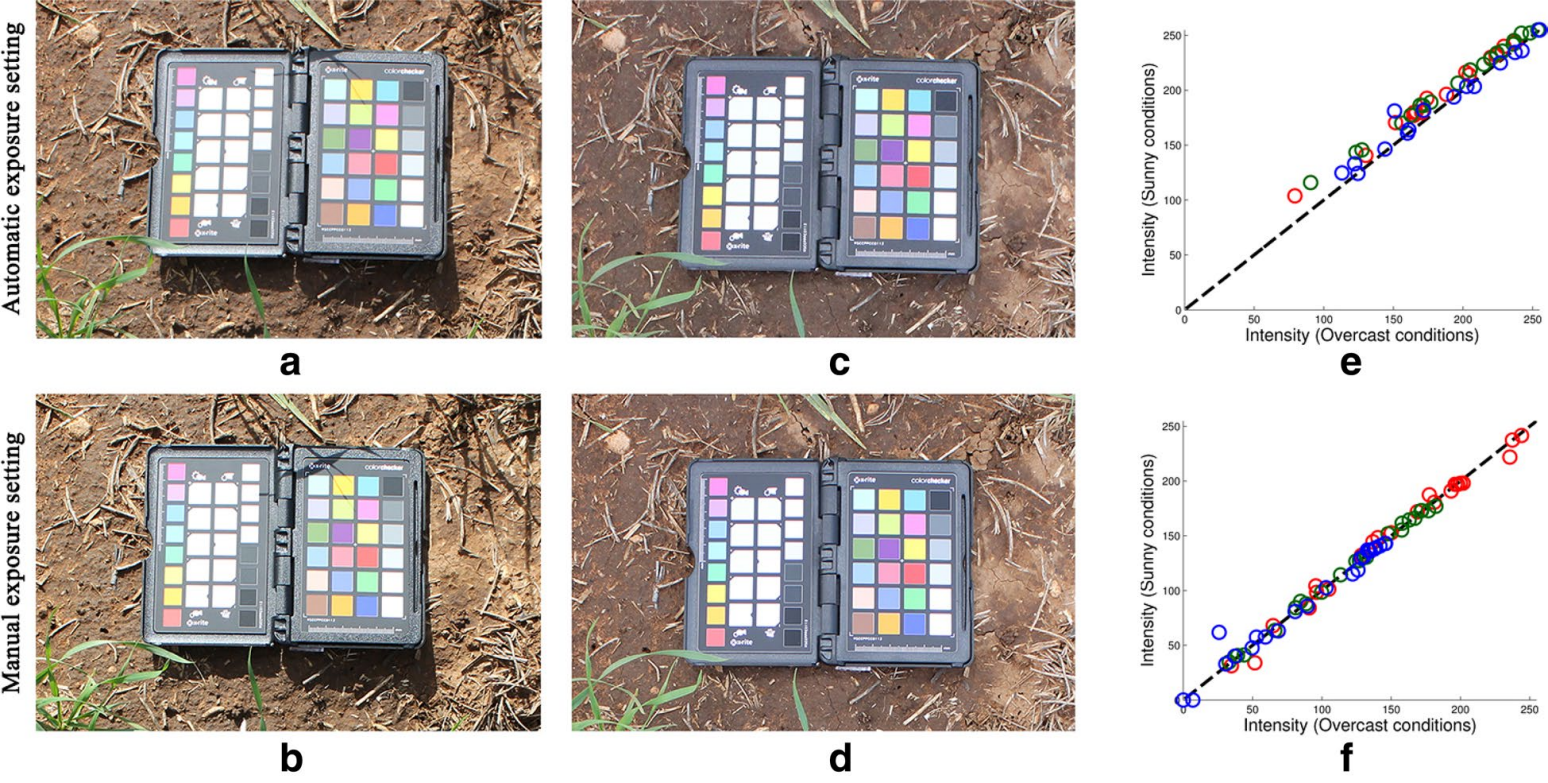
Manual versus automatic exposure settings. **a**, **b** Show images of the colour checker in the field under bright illumination, imaged with automatic and manual exposure settings, respectively. **c**, **d** Show images of the colour checker in the field during overcast conditions, imaged with automatic and manual exposure settings, respectively. **e**, **f** Show the difference in colour values after the illumination has varied when using automatic and manual exposure settings, without and with colour correction, respectively

Besides the artifacts affecting image quality, performing colour correction after imaging with a fixed exposure outperforms the use of auto-exposure features in terms of colour consistency. Figure 8 presents results of a short experiment comparing the two approaches. Sample images of the colour checker in well-lit field conditions taken with automatic and with manual exposure settings are shown in Fig. 8a, b, respectively. The same object is imaged in simulated overcast conditions, shown in Fig. 8c, d, using automatic and manual exposure settings, respectively. Figure 8e, f are plots showing the difference in colour values after illumination conditions have changed, when using automatic and manual exposure settings, without and with colour correction, respectively. It is clear that using automatic exposure settings has not completely corrected for the change in illumination, with all colour values having increased. In fact, the mean square error in colour values between the two illumination conditions is more than twice as large when using automatic exposure, 4.26, compared to manual exposure settings, 1.57.

### Colour corrected images for analysing variation in plant varieties and treatments

In this section we will use a number of colour corrected images to show the variations in canopy colour between different varieties, different treatments and different time periods that can be analysed. Note that Fig. 7 shows consistently small values in the blue channel, regardless of inconsistencies in illumination. This is due to plants reflecting larger amounts of red and green light than blue light in general. Due to this fact, hereinafter results will only be presented for the red and green channel, as they capture a large proportion of the variation in plot colour. Furthermore, a main part of this study will revolve around the two treatments applied to the plots, as a result they will hereinafter be abbreviated as fertilized (F) and non-fertilized (NF).

**Fig. 9:**
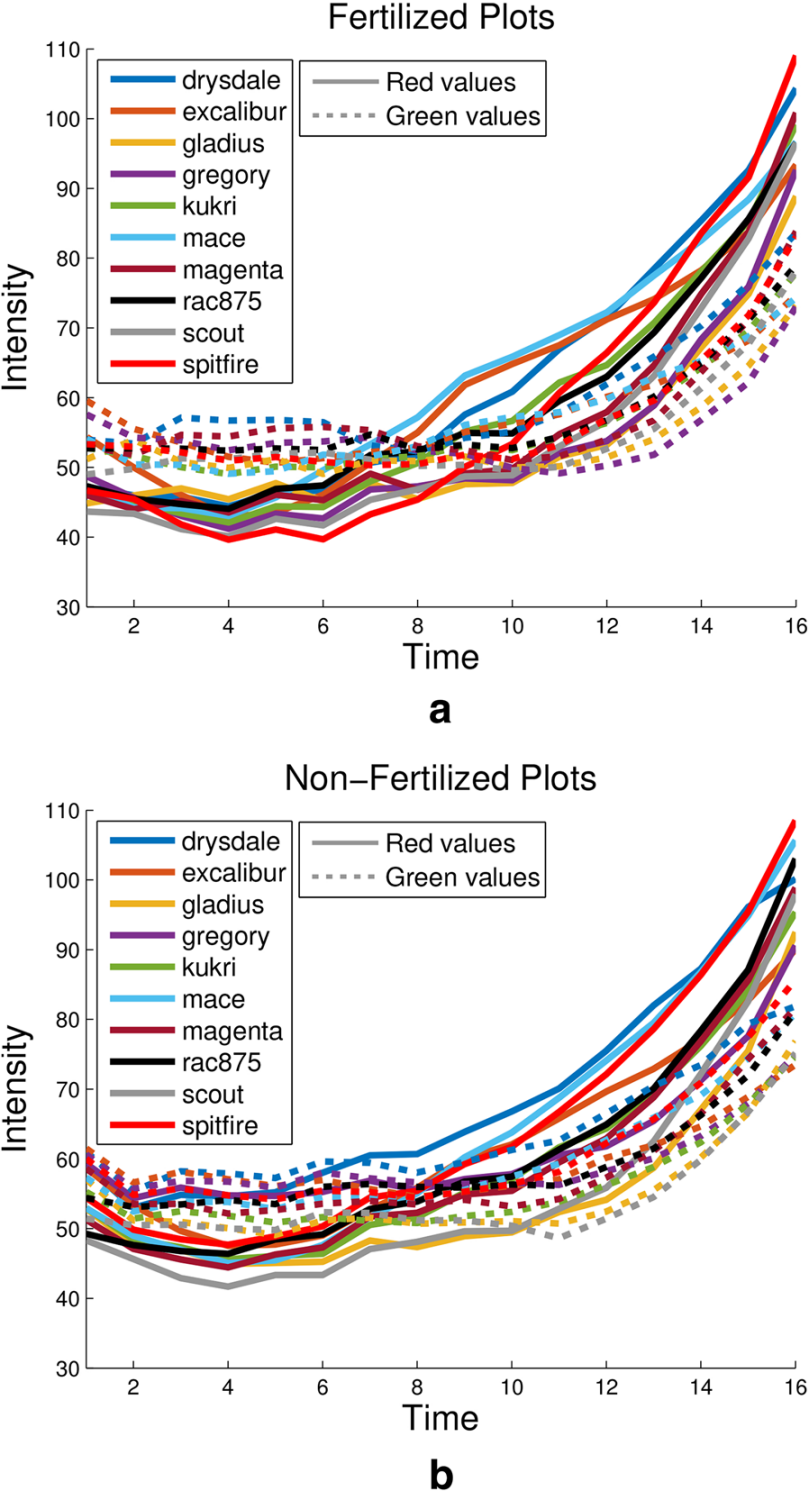
Mean intensities of all varieties, fertilized and non-fertilized. Plot showing the red and green values of ten wheat varieties both **a** fertilized and **b** non-fertilized for 16 imaging sessions. Different colours refer to different varieties, while dashed and solid lines refer to green and red values, respectively

The first result of this study, shown in Fig. 9, supports the hypothesis formed in the previous section, based on the contents of Fig. 7, that even under inconsistent field conditions colour corrected images are consistently able to exhibit the senescence stage of plants. Figure 9a shows that in the early, healthier, stages of plant life the green values of mean canopy colour are found almost exclusively above the red values, by a substantial margin, in fertilized plots. However toward the end of the time series the relationship is inverted and the intensity of red values is much higher. The same phenomenon can be seen in Fig. 9b, except here the separation between red and green values is not as distinct. Note that this is due to red values increasing in the NF plots, rather than green values decreasing, perhaps implying an early onset of decreased plant health.

**Fig. 10:**
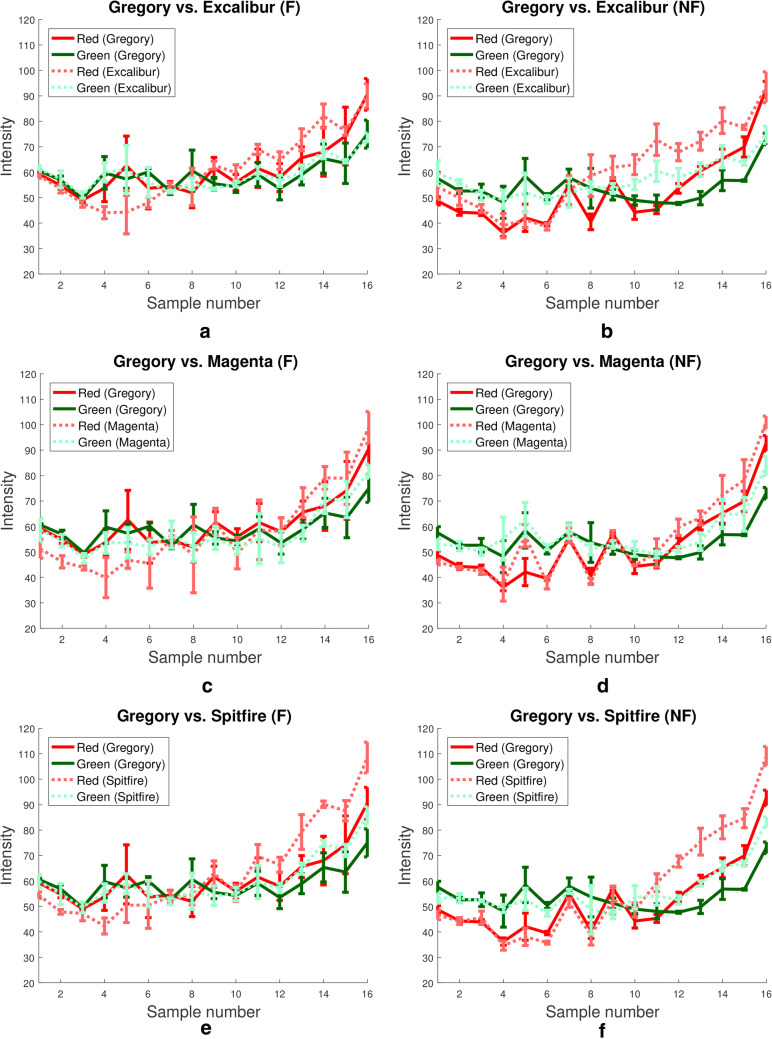
Corrected colour values for Gregory versus other varieties. Plots showing red and green mean intensities of images of plots of Gregory and Excalibur (**a** and **b**), Magenta (**c** and **d**) and Spitfire (**e** and **f**). Each row represents a different variety comparison with Gregory, while the left and right columns depict results for fertilised and non-fertilised plots, respectively

A range of post-harvest manual measurements were also obtained for the wheat plots grown in this study. The data from these measurements supports the hypothesis that mean plant canopy colour can be useful as a phenotypic trait that is related to other important traits such as yield. For example when averaging over the three replicates of each, the difference in total post-harvest biomass weight between F and NF plots of the same variety is 21.1 g. In the case of Gregory plots, the average difference in biomass between F and NF plots is 122 g. Similarly, the difference in straw weight between all F and NF plots is, on average, 26.5 g. However, for Gregory plots the difference is 82.7 g. Furthermore, Gregory appears to move through developmental stages at a slower rate than the other varieties, remaining on average more than 10 cm shorter than other varieties for most of the experiment.

We can support this manual data using the mean canopy colour information obtained from our colour corrected images. Figure 10 shows the red and green values for mean canopy colour over the length of the experiment for the Gregory, Excalibur, Magenta and Spitfire varieties. Each row shows the red and green values of Gregory plotted against the red and green values of a different variety and the two columns present the data of F and NF plots side by side. The first thing to note is that in all cases, including F and NF, the red values of Gregory are lower than the variety chosen for comparison. This implies that either Gregory is senescing significantly less than all other varieties, or, more plausibly, that it is taking longer to reach the stage of senescence, as the post-harvest data would also suggest. Furthermore, Gregory displayed the largest variation in green and red values between N and NF plots during early stages of growth. This can be seen from imaging sessions one to seven especially, where the red and green values in F plots are almost identical yet vary significantly in NF plots. This particular phenotypic trait could share a relationship with the biomass or straw weight which exhibited similar patterns.

**Fig. 11:**
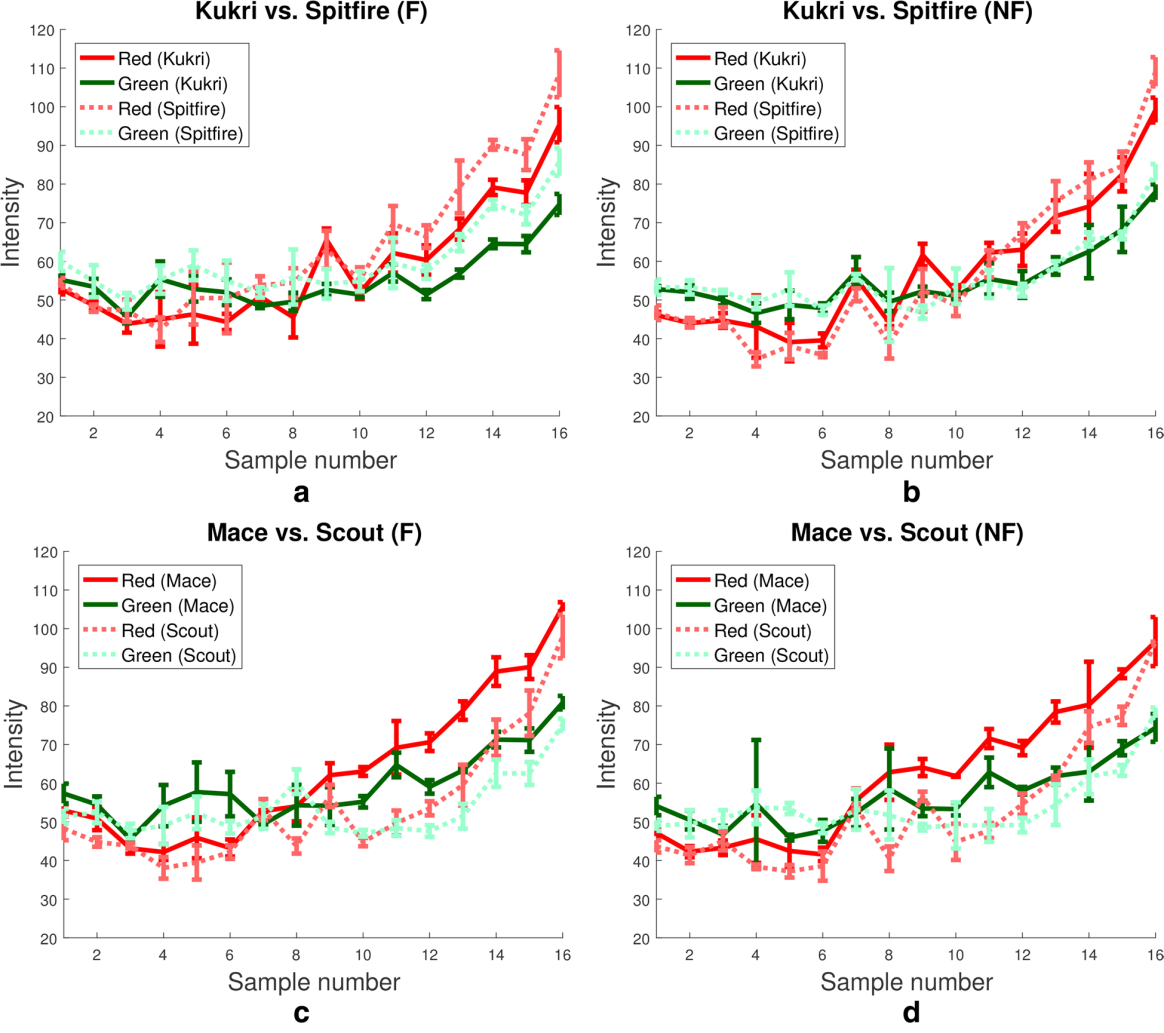
Colour values for plots containing different varieties. **a**, **b** Show plots of Kukri and Spitfire red and green values in fertilized and non-fertilized plots, respectively. **c**, **d** Show plots of Mace and Scout red and green values in fertilized and non-fertilized plots, respectively

Two more examples of variation between varieties and treatments, not involving the Gregory variety, are provided in Fig. 11. Figure 11a, b show that the varieties Kukri and Spitfire maintain very similar red and green values throughout all time points and both treatments, except for approximately the last third of time points in F plots where Spitfire exhibited much larger red and green values. Which could imply a significantly different reaction to nitrogen among two varieties which are otherwise very similar. In Fig. 11c, d we can see the converse, where the Mace and Scout varieties differ from each other in a similar pattern for both F and NF plots.

**Fig. 12:**
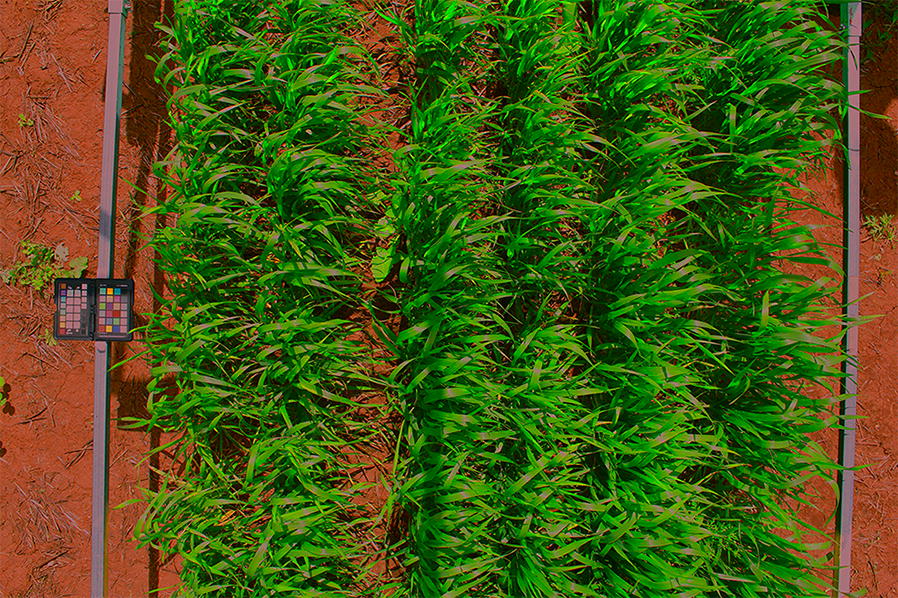
Sample image after inaccurate colour correction. In this image the colour checker is partially occluded by shadow. In rare cases the algorithm designed for detecting occlusions may fail, due to the manually defined threshold used for all images, resulting in an inaccurate measure of colour

One can also notice that the green values of the non-fertilized Mace variety on day 4 in Fig. 11d show an abnormally high standard deviation. In this case one of the three images of such plots contained a colour checker that was partially occluded and the algorithm designed for detecting occlusion failed. This particular plot is shown in Fig. 12, where one can see that part of the colour checker is occluded by shadow. Here the colour values of the tiles have been distorted enough to substantially alter the appearance of the image, but not enough to be detected by the threshold used for automatic occlusion detection. While this scenario only occurred a total of five times in more than 1000 test images, it serves as a reminder that clear and consistent imaging of the colour checker is paramount to final accuracy.

Finally, we provide an example of how to predict the NDVI of plants based on average canopy colour, in order to further demonstrate the importance and applicability of colour correcting a time series of plant images. A Trimble GreenSeeker [43] crop sensing system was used to manually measure the plant NDVI for all 60 plots on five select, approximately uniformly spaced, days of the experiment. NDVI has been used to estimate crop yield and is also directly related to other phenotypic traits such as photosynthetic activity and biomass. Equation 3 provides the formula for calculating plant NDVI, where NIR and R denote the near-infrared and red values recorded by the GreenSeeker system, respectively.

3 $$\begin{aligned} \hbox {NDVI} = \frac{NIR - R}{NIR + R}, \end{aligned}$$

This comparison is especially useful as NDVI values are independent of illumination conditions, since the sum in the denominator of Eq. 3 ensures that plant patches that are more illuminated are scaled down in magnitude. The first 4 days are used as training data, where the average canopy colour and NDVI of each plot are known. Using the mean canopy colour of both corrected and uncorrected images, two quadratic models are fitted to the NDVI data using the same approach outlined in the Methods section. The statistical method is then used to predict the NDVI values from plant images taken on the fifth day. This is essentially the scheme proposed by Khan et al. [44].

**Fig. 13:**
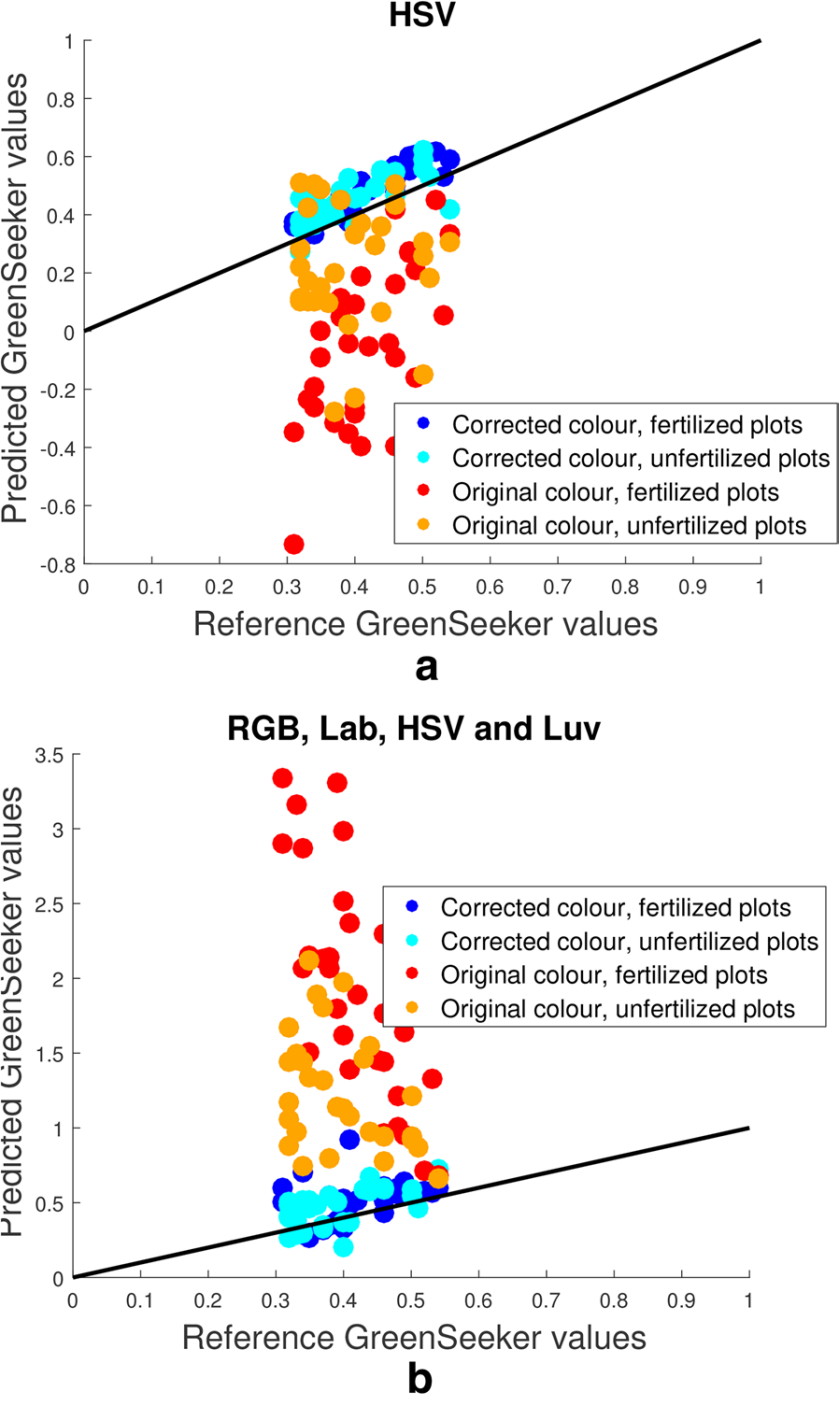
Predicting GreenSeeker values using mean canopy colour. A quadratic model has been used to predict GreenSeeker values recorded from all 60 plots. The black line represents perfect prediction, where reference values would match predicted values exactly. Light and dark blue circles represent where colour corrected images were used as training data for the prediction model, orange and red circles refer to the uncorrected images. **a** Using mean canopy colour in the HSV colour space for prediction. **b** Using mean canopy colour in RGB, Lab, HSV and Luv colour spaces for prediction

Figure 13 shows the reference NDVI values in the horizontal axis and the predicted NDVI values in the vertical axis, for cases with two different colour spaces used as training data. Figure 13a used values from the HSV colour space to predict NDVI values, while Fig. 13b used data from the RGB, HSV, Lab and Luv colour spaces. In both cases it is clear that the training data consisting of colour corrected images, blue circles, has more accurately predicted NDVI values than the uncorrected images, red and orange circles. To quantify the accuracy of prediction we again utilise the MSE to determine error, which in this case is calculated as

4 $$\begin{aligned} E = \frac{1}{N} \sum _{i=1}^{N} \left( \widehat{NDVI}_i - NDVI_i \right) ^2, \end{aligned}$$

where *N* is the number of samples (60 in this case) and $\widehat{NDVI}_i$ and $NDVI_i$ are the reference and observed NDVI values for the *i*th plot, respectively. Table 3 shows the results of using different colour channels as training data. The table shows that in all cases the corrected mean canopy colour was more accurate than the uncorrected data in predicting the true NDVI values, sometimes by as much as an order of magnitude. It is worth noting that Fig. 13a, b appear to further suggest a relationship between a plot’s mean canopy colour and fertilization of the plot. A further study could be employed to determine the accuracy in predicting whether or not a plot has been fertilized, based on its mean canopy colour. Preliminary results show that this type of study can also provide insight into the behaviour of different varieties. As previously stated, the variety Gregory showed the slowest development of all varieties and a significant difference in mean canopy colour between fertilised and non-fertilised plots. In the NDVI study, predicted and reference NDVI values both showed that Gregory plots contained the greatest difference in mean NDVI values between fertilised and non-fertilised plots.

**Table 3:** Mean square error (*MSE*) values for predicting plant NDVI values using the mean canopy colour of different colour spaces, before and after colour correction (CC)

	RGB	HSV	Lab	Luv	All
Before CC	0.487	0.066	0.177	0.032	1.901
After CC	0.012	0.014	0.006	0.008	0.019

## Conclusions

The scope of plant phenotyping has expanded to include image-based analysis of the field. While increased capabilities increase the potential for phenomics experiments they also require more advanced methods of analysis. In this article we have proposed an approach for ascertaining the important trait of plant canopy colour in a correct, accurate and robust manner. We have shown that using an industry standard colour checker and a quadratic model for colour correction we are able to establish a consistent assessment of colour condition irresepctive of fluctuating illumination. We have demonstrated that by performing accurate colour correction we provide an accurate and robust measure of mean canopy colour, a useful phenotypic trait which may be directly related to plant NDVI, but also provides a method for consistently measuring colour over time and over varying levels of illumination. In future work we shall consider the role that colour correction plays on detecting and quantifying plant sensecence in the field.

## Additional files

**Additional file 1.** Image processing. **Figure S1**. Extracting the region of interest. First the rails of the wagon from the original image are detected using a combination of grayscale thresholding, Hough transforms and a number of morphological operations. The image is then ‘clipped’ to contain only the region of interest, hence removing the possibility of weeds or the colour checker being detected as foreground. **Figure S2**. Support Vector Machine. The images are segmented using the pictured support vector machine classifier which was trained on the output of the k-means clustering algorithm. The x and y axes are u and v values, respectively, from the Luv colour space. The gray and purple regions represent the green plant class and background class, respectively. The green and black circles represent training data of green plant pixel values and background pixel values, respectively.
