# Supplementary material for: Land-based crop phenotyping by image analysis: consistent canopy characterization from inconsistent field illumination
Source: Plant Methods. 2018 May 26;14:39. doi: 10.1186/s13007-018-0308-5 (PMC5970541; doi:10.1186/s13007-018-0308-5)
Supplement: Supplementary file 1 — Additional file 1. Image processing. Figure S1. Extracting the region of interest. First the rails of the wagon from the original image are detected using a combination of grayscale thresholding, Hough transforms and a number of morphological operations. The image is then ‘clipped’ to contain only the region of interest, hence removing the possibility of weeds or the colour checker being detected as foreground. Figure S2. Support Vector Machine. The images are segmented using the pictured support vector machine classifier which was trained on the output of the k-means clustering algorithm. The x and y axes are u and v values, respectively, from the Luv colour space. The gray and purple regions represent the green plant class and background class, respectively. The green and black circles represent training data of green plant pixel values and background pixel values, respectively. [file 13007_2018_308_MOESM1_ESM.pdf]

Land-based crop phenotyping by image analysis: consistent canopy characterization from inconsistent field illumination

Additional File 1: Image processing

Chopin, Kumar, Miklavcic

Image pre-processing

Region of interest detection.

Due to transport over the inhomogeneous field terrain, the camera positions change slightly between plots. This affects where plants lie in the image. Hence, the region of interest needs to be calculated individually for each image.The steel rails contain the majority of the brightest pixels in the image. Hence, calculating the Euclidean distance between the colour of every pixel in a given image, $I \in [0, 255]^3$, and the colour white (full intensity) provides an initial estimate of the rail locations. The new image $\hat{I} = |255 - I|$ is then thresholded so that all pixels with $\hat{I} \leq 0.1$ are classified as belonging to the vehicle rails. The foreground of the new binary image consists mostly of rail pixels but also a significant amount of noise from plant leaves reflecting light or various colour checker tiles. To remove this noise the Hough transform is used to detect and keep groups of foreground pixels that correspond to straight lines. The straight lines detected in a sample image can be seen in the centre frame of Figure S1. The two parallel rails are then used to create the left and right boundaries of a binary quadrilateral mask, whose upper and lower edges are simply the top and bottom rows of image pixels. Finally, the Hadamard product of the image and the binary mask results in an image containing only the region of interest, here shown in Figure S1.

Colour chart extraction. Colour values from the individual tiles of the colour checker need to be recorded from each image, for subsequent colour correction. Potential occlusions from plant leaves and the large variation in illumination within a day and over multiple days make this a challenging task. To locate the colour chart in each image a basic template

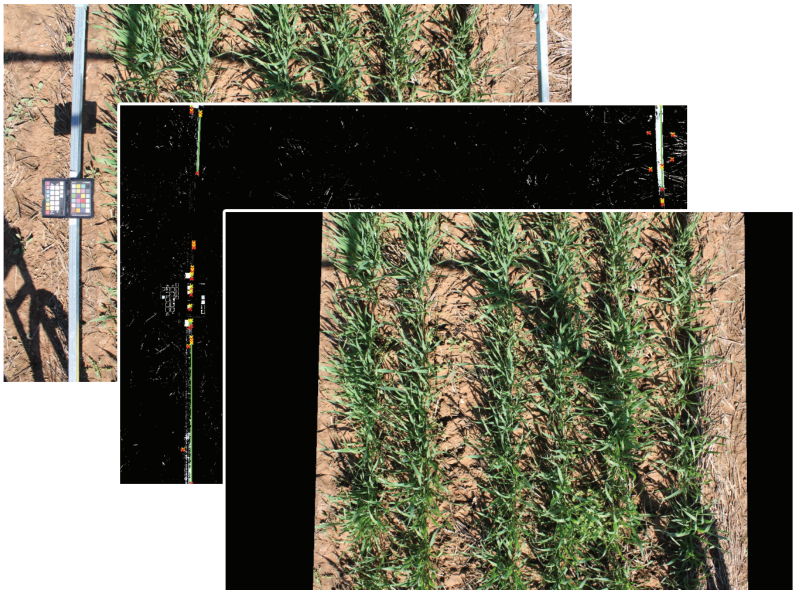

Figure S1 Extracting the region of interest. First the rails of the wagon from the original image are detected using a combination of grayscale thresholding, hough transforms and a number of morphological operations. The image is then 'clipped' to contain only the region of interest, hence removing the possibility of weeds or the colour checker being detected as foreground.

matching algorithm is used. A template image of the colour chart is chosen and its centre is passed iteratively over every pixel in the image. The Euclidean distance between the red, green and blue values of the $m \times n$ template and corresponding $m \times n$ region of the original image is then calculated, where the minimum value over I is chosen to be the centre of the colour chart. To reduce computational time the template can be applied to a smaller subset of the image known to contain the colour chart. Once the centre of the colour chart is located, the red, green and blue values of individual tiles are stored for each image. A short algorithm is then used in order to reduce the risk of a tile occluded by plant leaves being used in the colour correction stage. For a given day, or imaging session, the average red, $\bar{r}_k$, green, $\bar{g}_k$ and blue, $\bar{b}_k$, values for tile k are recorded. If the red, green or blue values for tile k, taken from the i-th plot, differs from tile k's mean value by more than a predefined threshold (i.e. $r_k^i, g_k^i, b_k^i \geq T$), that tile is said to be occluded by outside sources, such as a

plant leaf. The colour values for the non-occluded tile in the image closest chronologically is used in place of the occluded tile values.

Segmentation

Plant pixels in all images were segmented from the background using support vector machines (SVM) which were trained on the output of k-means clustering. SVM is a supervised machine learning technique which, for a set of data with two classes, attempts to find the best hyperplane that separates the two data classes. The data points closest to the hyperplane are called the support vectors. Minimizing the distance between them determines which hyperplane is 'best'. There are many options to choose from when selecting data to train for the classifier, as we have access to multiple colour spaces with three channels each. In this work we found the u and v channels of the Luv colour space the most effective for segmenting plant pixels from the image background. Figure S2 shows the training data used to train our support vector machine, with green and black circles representing plant and background pixel values, respectively. As the boundary between the two regions appears to be non-linear, a radial basis function (RBF) kernel was chosen rather than a polynomial. The SVM created with the RBF is illustrated by the grey and purple regions, which represent regions where new pixels would be classified as plant or background, respectively. Generally, in the literature, SVMs have been trained on a number of entire segmented images. However, the process of manually segmenting every pixel from a moderate to large number of images for training purposes is tedious. Instead, we make use of the k-means clustering algorithm for training. Using k-means clustering, each training image is segmented into 20 clusters with minimal intra-class variance, then each cluster is given a label as green plant or background. The centre of each cluster, or mean colour, is then used as training data for the SVM, represented by the green and black circles in Figure S2. As this process takes far less time than manually segmenting entire images, it allows more total images to be used for training, capturing more variation across plots and over time.

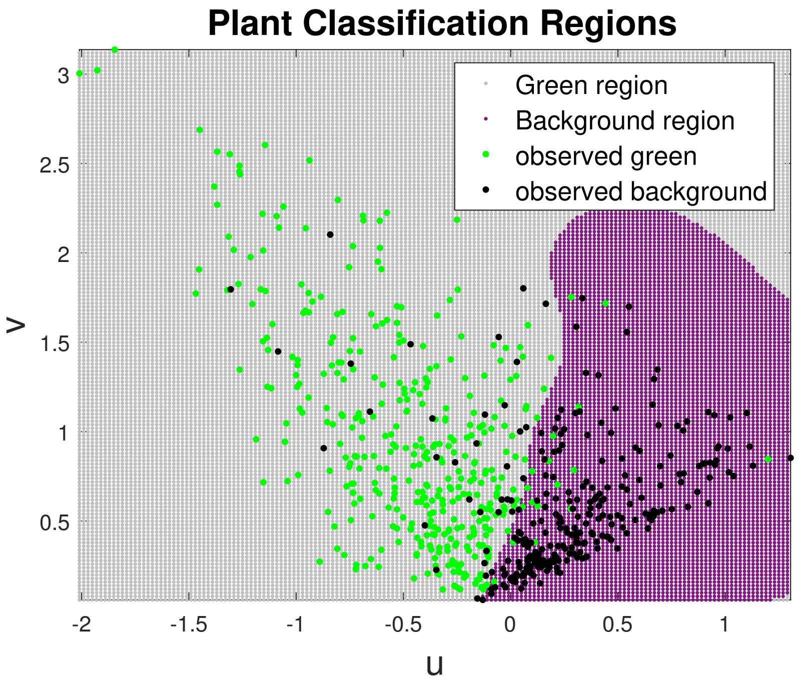

Figure S2 Support Vector Machine. The images are segmented using the pictured support vector machine classifier which was trained on the output of the k-means clustering algorithm. The x and y axes are u and v values, respectively, from the Luv colour space. The gray and purple regions represent the green plant class and background class, respectively. The green and black circles represent training data of green plant pixel values and background pixel values, respectively.
